# 4-[(1,5-Dibenzyl-2,4-dioxo-2,3,4,5-tetra­hydro-1*H*-1,5-benzodiazepin-3-yl)meth­yl]-1-*n*-octyl-1*H*-1,2,3-triazole

**DOI:** 10.1107/S1600536809054476

**Published:** 2009-12-24

**Authors:** Hind Jabli, F. Ouazzani Chahdi, Natalie Saffon, El Mokhtar Essassi, Seik Weng Ng

**Affiliations:** a Laboratoire de Chimie Organique Appliquée, Faculté des Sciences et Techniques, Université Sidi Mohamed Ben Abdallah, Fés, Morocco; b Service Commun Rayons-X FR2599, Université Paul Sabatier, Bâtiment 2R1, 118 Route de Narbonne, Toulouse, France; c Laboratoire de Chimie Organique Hétérocyclique, Pôle de Compétences Pharmacochimie, Université Mohammed V-Agdal, BP 1014 Avenue Ibn Batout, Rabat, Morocco; d Department of Chemistry, University of Malaya, 50603 Kuala Lumpur, Malaysia

## Abstract

The reaction of 1,5-dibenzyl-3-propargyl-1,5-benzodiazepine-2,4-dione with 1-azido-*n*-octane in the presence of catalysts leads to the formation of the title compound, C_34_H_39_N_5_O_2_, which features a phenyl­ene ring fused with a seven-membered diazepinyl ring. The latter ring adopts a boat conformation with the octyltriazolylmethyl-bearing C atom as the prow and the fused-ring C atoms as the stern. The octyltriazolylmethyl substituent occupies an axial position.

## Related literature

For the crystal structures of other *N*-substituted homologues, see: Jabli *et al.* (2009 ▶, 2010 ▶).
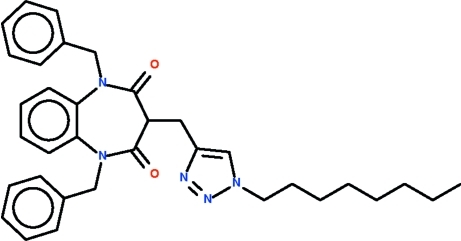


## Experimental

### 

#### Crystal data

C_34_H_39_N_5_O_2_
*M* *_r_* = 549.70
Orthorhombic, 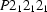

*a* = 9.1797 (2) Å
*b* = 10.4238 (2) Å
*c* = 31.4716 (7) Å
*V* = 3011.4 (1) Å^3^
*Z* = 4
Mo *K*α radiation
μ = 0.08 mm^−1^
*T* = 193 K
0.30 × 0.16 × 0.10 mm

#### Data collection

Bruker APEXII diffractometer
44767 measured reflections
3027 independent reflections
2307 reflections with *I* > 2σ(*I*)
*R*_int_ = 0.072

#### Refinement

*R*[*F* ^2^ > 2σ(*F* ^2^)] = 0.057
*wR*(*F* ^2^) = 0.174
*S* = 1.02
3027 reflections
370 parameters
59 restraints
H-atom parameters constrained
Δρ_max_ = 0.59 e Å^−3^
Δρ_min_ = −0.41 e Å^−3^

### 

Data collection: *APEX2* (Bruker, 2005 ▶); cell refinement: *SAINT* (Bruker, 2005 ▶); data reduction: *SAINT*; program(s) used to solve structure: *SHELXS97* (Sheldrick, 2008 ▶); program(s) used to refine structure: *SHELXL97* (Sheldrick, 2008 ▶); molecular graphics: *X-SEED* (Barbour, 2001 ▶); software used to prepare material for publication: *publCIF* (Westrip, 2010 ▶).

## Supplementary Material

Crystal structure: contains datablocks global, I. DOI: 10.1107/S1600536809054476/bt5142sup1.cif

Structure factors: contains datablocks I. DOI: 10.1107/S1600536809054476/bt5142Isup2.hkl

Additional supplementary materials:  crystallographic information; 3D view; checkCIF report

## Figures and Tables

**Fig. 1.**
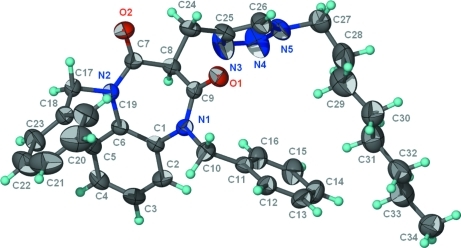
Anisotropic displacement ellipsoid plot (Barbour, 2001) of C34H23N5O2 at the 70% probability level; hydrogen atoms are drawn as arbitrary radius.

## supplementary crystallographic information

### Experimental

To a solution 1,5-dibenzyl-3-propargyl-1,5-benzodiazepine-2,4-dione (1 mmol) *t*-butyl alcohol/water (1/2, 8 ml) was added copper sulfate pentahydrate (1 mmol), sodium ascorbate (2 mmol) and 1-azido-*n*-octane (5 mmol). Stirring was continued for 12 h. The solution was diluted with water (20 ml) and the organic compound extracted with ethyl acetate (2 *x* 20 ml). The extracts were washed with brine and dried over sodium sulfate. The compound was recrystallized from ethyl acetate/ether to give colorless crystals.

### Refinement

Carbon-bound H-atoms were placed in calculated positions (C—H 0.95 to 0.99 Å) and were included in the refinement in the riding model approximation, with *U*(H) set to 1.2–1.5*U*(C).

For the octyl chain, the 1,2-related carbon-carbon distances were restrained to 1.54±0.01 Å and the 1,3-related ones to 2.51±0.01 Å. The anisotropic displacement ellipsoids were restrained to be nearly isotropic.

### Crystal data

**Table:**

C_34_H_39_N_5_O_2_	*F*(000) = 1176
*M**_r_* = 549.70	*D*_x_ = 1.212 Mg m^−^^3^
Orthorhombic, *P*2_1_2_1_2_1_	Mo *K*α radiation, λ = 0.71073 Å
Hall symbol: P 2ac 2ab	Cell parameters from 5123 reflections
*a* = 9.1797 (2) Å	θ = 2.3–19.1°
*b* = 10.4238 (2) Å	µ = 0.08 mm^−^^1^
*c* = 31.4716 (7) Å	*T* = 193 K
*V* = 3011.4 (1) Å^3^	Block, colorless
*Z* = 4	0.30 × 0.16 × 0.10 mm

### Data collection

**Table:**

Bruker APEXII diffractometer	2307 reflections with *I* > 2σ(*I*)
Radiation source: fine-focus sealed tube	*R*_int_ = 0.072
graphite	θ_max_ = 25.0°, θ_min_ = 1.3°
φ and ω scans	*h* = −10→10
44767 measured reflections	*k* = −11→12
3027 independent reflections	*l* = −37→37

### Refinement

**Table:**

Refinement on *F*^2^	Secondary atom site location: difference Fourier map
Least-squares matrix: full	Hydrogen site location: inferred from neighbouring sites
*R*[*F*^2^ > 2σ(*F*^2^)] = 0.057	H-atom parameters constrained
*wR*(*F*^2^) = 0.174	*w* = 1/[σ^2^(*F*_o_^2^) + (0.1036*P*)^2^ + 1.5833*P*] where *P* = (*F*_o_^2^ + 2*F*_c_^2^)/3
*S* = 1.02	(Δ/σ)_max_ = 0.001
3027 reflections	Δρ_max_ = 0.59 e Å^−^^3^
370 parameters	Δρ_min_ = −0.40 e Å^−^^3^
59 restraints	Absolute structure: Friedel pairs were merged
Primary atom site location: structure-invariant direct methods	

### Fractional atomic coordinates and isotropic or equivalent isotropic displacement parameters (Å^2^)

**Table:**

	*x*	*y*	*z*	*U*_iso_*/*U*_eq_	
O1	0.6191 (3)	0.4931 (3)	0.14072 (10)	0.0418 (8)	
O2	0.6411 (3)	0.6919 (4)	0.04134 (9)	0.0449 (8)	
N1	0.8576 (4)	0.4659 (3)	0.12497 (11)	0.0316 (8)	
N2	0.8703 (4)	0.6098 (3)	0.04556 (11)	0.0312 (8)	
N3	0.7316 (5)	0.8647 (5)	0.18835 (15)	0.0663 (14)	
N4	0.7049 (5)	0.8835 (5)	0.22909 (16)	0.0658 (15)	
N5	0.5823 (5)	0.8206 (4)	0.23825 (13)	0.0453 (10)	
C1	0.9921 (5)	0.5125 (4)	0.10829 (13)	0.0306 (10)	
C2	1.1212 (5)	0.4862 (5)	0.12995 (15)	0.0383 (11)	
H2	1.1178	0.4408	0.1561	0.046*	
C3	1.2544 (5)	0.5254 (5)	0.11379 (16)	0.0434 (12)	
H3	1.3415	0.5062	0.1288	0.052*	
C4	1.2608 (5)	0.5919 (5)	0.07613 (16)	0.0432 (12)	
H4	1.3524	0.6176	0.0650	0.052*	
C5	1.1341 (5)	0.6214 (4)	0.05445 (14)	0.0356 (10)	
H5	1.1393	0.6689	0.0287	0.043*	
C6	0.9983 (5)	0.5823 (4)	0.06998 (13)	0.0295 (10)	
C7	0.7468 (5)	0.6614 (4)	0.06302 (13)	0.0323 (10)	
C8	0.7454 (5)	0.6736 (4)	0.11111 (13)	0.0327 (10)	
H8	0.8395	0.7117	0.1209	0.039*	
C9	0.7325 (5)	0.5370 (4)	0.12750 (13)	0.0312 (10)	
C10	0.8521 (5)	0.3333 (4)	0.14053 (13)	0.0355 (10)	
H10A	0.9323	0.2846	0.1270	0.043*	
H10B	0.7593	0.2944	0.1310	0.043*	
C11	0.8638 (5)	0.3163 (4)	0.18838 (13)	0.0325 (9)	
C12	0.8477 (5)	0.1925 (5)	0.20461 (14)	0.0386 (11)	
H12	0.8293	0.1228	0.1859	0.046*	
C13	0.8583 (6)	0.1711 (5)	0.24805 (16)	0.0488 (13)	
H13	0.8454	0.0869	0.2590	0.059*	
C14	0.8876 (6)	0.2714 (6)	0.27544 (17)	0.0535 (14)	
H14	0.8957	0.2567	0.3051	0.064*	
C15	0.9048 (6)	0.3930 (6)	0.25920 (16)	0.0522 (14)	
H15	0.9262	0.4622	0.2779	0.063*	
C16	0.8913 (5)	0.4160 (5)	0.21615 (14)	0.0411 (11)	
H16	0.9010	0.5010	0.2056	0.049*	
C17	0.8803 (5)	0.6010 (4)	−0.00113 (12)	0.0335 (10)	
H17A	0.9558	0.6615	−0.0112	0.040*	
H17B	0.7861	0.6278	−0.0136	0.040*	
C18	0.9170 (5)	0.4679 (5)	−0.01692 (14)	0.0358 (10)	
C19	0.8245 (6)	0.3651 (5)	−0.00935 (19)	0.0567 (15)	
H19	0.7407	0.3772	0.0079	0.068*	
C20	0.8518 (8)	0.2465 (6)	−0.0263 (2)	0.078 (2)	
H20	0.7872	0.1770	−0.0211	0.093*	
C21	0.9754 (8)	0.2290 (6)	−0.0514 (2)	0.0731 (19)	
H21	0.9946	0.1477	−0.0638	0.088*	
C22	1.0674 (7)	0.3274 (7)	−0.05782 (19)	0.0701 (18)	
H22	1.1529	0.3146	−0.0743	0.084*	
C23	1.0392 (6)	0.4459 (6)	−0.04097 (15)	0.0476 (13)	
H23	1.1055	0.5142	−0.0460	0.057*	
C24	0.6185 (5)	0.7586 (5)	0.12581 (14)	0.0420 (11)	
H24A	0.6194	0.8394	0.1092	0.050*	
H24B	0.5255	0.7141	0.1198	0.050*	
C25	0.6250 (5)	0.7901 (4)	0.17170 (14)	0.0381 (11)	
C26	0.5306 (5)	0.7603 (5)	0.20384 (15)	0.0438 (12)	
H26	0.4462	0.7079	0.2021	0.053*	
C27	0.5207 (7)	0.8285 (6)	0.28125 (16)	0.0652 (16)	
H27A	0.4189	0.7973	0.2803	0.078*	
H27B	0.5181	0.9198	0.2900	0.078*	
C28	0.6026 (10)	0.7531 (7)	0.3147 (2)	0.104 (3)	
H28A	0.7003	0.7919	0.3182	0.125*	
H28B	0.5507	0.7625	0.3421	0.125*	
C29	0.6209 (9)	0.6171 (6)	0.3060 (2)	0.099 (2)	
H29A	0.6933	0.6065	0.2830	0.119*	
H29B	0.5271	0.5817	0.2958	0.119*	
C30	0.6720 (7)	0.5384 (6)	0.3458 (2)	0.0790 (19)	
H30A	0.5954	0.5441	0.3679	0.095*	
H30B	0.6807	0.4471	0.3375	0.095*	
C31	0.8145 (6)	0.5805 (6)	0.36514 (19)	0.0740 (19)	
H31A	0.7996	0.6630	0.3801	0.089*	
H31B	0.8861	0.5951	0.3421	0.089*	
C32	0.8765 (7)	0.4832 (7)	0.39625 (19)	0.0773 (19)	
H32A	0.9042	0.4045	0.3806	0.093*	
H32B	0.8004	0.4597	0.4172	0.093*	
C33	1.0108 (7)	0.5354 (8)	0.4200 (2)	0.088 (2)	
H33A	1.0927	0.5444	0.3998	0.105*	
H33B	0.9881	0.6216	0.4314	0.105*	
C34	1.0553 (8)	0.4517 (8)	0.4550 (2)	0.086 (2)	
H34A	1.1417	0.4876	0.4689	0.129*	
H34B	1.0782	0.3662	0.4438	0.129*	
H34C	0.9758	0.4450	0.4756	0.129*	

### Atomic displacement parameters (Å^2^)

**Table:**

	*U*^11^	*U*^22^	*U*^33^	*U*^12^	*U*^13^	*U*^23^
O1	0.0256 (16)	0.055 (2)	0.0451 (18)	−0.0050 (16)	0.0030 (14)	0.0068 (15)
O2	0.0338 (17)	0.066 (2)	0.0345 (16)	0.0123 (18)	−0.0067 (15)	0.0071 (16)
N1	0.0299 (19)	0.0324 (19)	0.0325 (18)	0.0024 (17)	0.0018 (16)	0.0069 (16)
N2	0.0281 (18)	0.038 (2)	0.0270 (18)	0.0016 (18)	−0.0004 (16)	0.0033 (15)
N3	0.061 (3)	0.084 (4)	0.054 (3)	−0.025 (3)	0.014 (2)	−0.012 (3)
N4	0.058 (3)	0.078 (4)	0.062 (3)	−0.026 (3)	0.011 (2)	−0.026 (3)
N5	0.039 (2)	0.054 (3)	0.043 (2)	0.005 (2)	0.0033 (18)	−0.009 (2)
C1	0.027 (2)	0.034 (2)	0.031 (2)	0.003 (2)	0.0016 (18)	0.0002 (19)
C2	0.032 (2)	0.044 (3)	0.039 (2)	0.007 (2)	−0.003 (2)	0.005 (2)
C3	0.029 (2)	0.050 (3)	0.051 (3)	0.007 (2)	−0.003 (2)	0.002 (3)
C4	0.028 (2)	0.050 (3)	0.052 (3)	0.000 (2)	0.007 (2)	0.000 (3)
C5	0.028 (2)	0.039 (2)	0.039 (2)	−0.002 (2)	0.002 (2)	0.002 (2)
C6	0.026 (2)	0.032 (2)	0.030 (2)	0.004 (2)	0.0005 (18)	−0.0012 (18)
C7	0.028 (2)	0.038 (2)	0.031 (2)	0.003 (2)	0.0000 (18)	0.004 (2)
C8	0.025 (2)	0.038 (2)	0.035 (2)	0.005 (2)	0.0011 (18)	0.000 (2)
C9	0.031 (2)	0.039 (2)	0.024 (2)	0.003 (2)	0.0012 (18)	0.0041 (19)
C10	0.040 (2)	0.031 (2)	0.035 (2)	−0.001 (2)	−0.004 (2)	0.0020 (19)
C11	0.025 (2)	0.035 (2)	0.037 (2)	0.001 (2)	0.0014 (19)	0.0009 (19)
C12	0.041 (3)	0.034 (2)	0.041 (3)	0.000 (2)	0.002 (2)	0.004 (2)
C13	0.044 (3)	0.049 (3)	0.053 (3)	0.003 (3)	−0.004 (2)	0.020 (3)
C14	0.049 (3)	0.072 (4)	0.040 (3)	−0.003 (3)	−0.003 (2)	0.012 (3)
C15	0.059 (3)	0.059 (3)	0.039 (3)	−0.011 (3)	−0.004 (2)	−0.003 (2)
C16	0.044 (3)	0.040 (3)	0.039 (3)	−0.007 (2)	0.004 (2)	0.002 (2)
C17	0.033 (2)	0.040 (2)	0.028 (2)	0.002 (2)	0.0015 (19)	0.0038 (18)
C18	0.033 (2)	0.042 (3)	0.032 (2)	−0.001 (2)	−0.0010 (19)	0.002 (2)
C19	0.053 (3)	0.041 (3)	0.076 (4)	−0.004 (3)	0.009 (3)	0.000 (3)
C20	0.074 (4)	0.047 (3)	0.112 (6)	−0.005 (4)	−0.011 (4)	−0.005 (4)
C21	0.078 (5)	0.054 (4)	0.088 (5)	0.020 (4)	−0.020 (4)	−0.027 (3)
C22	0.062 (4)	0.084 (5)	0.064 (4)	0.019 (4)	0.008 (3)	−0.023 (4)
C23	0.042 (3)	0.057 (3)	0.043 (3)	0.006 (3)	0.007 (2)	−0.008 (3)
C24	0.032 (2)	0.050 (3)	0.044 (3)	0.013 (2)	−0.001 (2)	−0.003 (2)
C25	0.027 (2)	0.040 (3)	0.047 (3)	0.006 (2)	0.004 (2)	−0.005 (2)
C26	0.036 (3)	0.052 (3)	0.043 (3)	−0.005 (2)	0.004 (2)	−0.005 (2)
C27	0.065 (3)	0.087 (4)	0.044 (3)	0.000 (4)	0.004 (3)	−0.012 (3)
C28	0.113 (6)	0.116 (6)	0.083 (5)	0.011 (5)	0.017 (5)	0.006 (5)
C29	0.082 (5)	0.112 (6)	0.103 (5)	0.006 (5)	−0.004 (4)	−0.023 (5)
C30	0.071 (4)	0.072 (4)	0.094 (5)	−0.003 (4)	−0.002 (4)	0.007 (4)
C31	0.076 (4)	0.063 (4)	0.083 (4)	−0.018 (3)	0.020 (3)	−0.016 (3)
C32	0.070 (4)	0.090 (4)	0.072 (4)	−0.017 (4)	0.014 (3)	−0.001 (3)
C33	0.078 (4)	0.091 (5)	0.093 (5)	−0.015 (4)	0.008 (4)	0.013 (4)
C34	0.068 (4)	0.091 (5)	0.099 (5)	−0.006 (4)	0.001 (4)	0.019 (4)

### Geometric parameters (Å, °)

**Table:**

O1—C9	1.211 (5)	C17—H17A	0.9900
O2—C7	1.228 (5)	C17—H17B	0.9900
N1—C9	1.369 (6)	C18—C23	1.373 (6)
N1—C1	1.426 (5)	C18—C19	1.388 (7)
N1—C10	1.468 (6)	C19—C20	1.369 (8)
N2—C7	1.370 (6)	C19—H19	0.9500
N2—C6	1.433 (5)	C20—C21	1.393 (10)
N2—C17	1.475 (5)	C20—H20	0.9500
N3—N4	1.320 (6)	C21—C22	1.344 (10)
N3—C25	1.356 (6)	C21—H21	0.9500
N4—N5	1.334 (6)	C22—C23	1.368 (9)
N5—C26	1.339 (6)	C22—H22	0.9500
N5—C27	1.469 (6)	C23—H23	0.9500
C1—C2	1.394 (6)	C24—C25	1.482 (6)
C1—C6	1.409 (6)	C24—H24A	0.9900
C2—C3	1.386 (7)	C24—H24B	0.9900
C2—H2	0.9500	C25—C26	1.367 (6)
C3—C4	1.374 (7)	C26—H26	0.9500
C3—H3	0.9500	C27—C28	1.513 (7)
C4—C5	1.384 (7)	C27—H27A	0.9900
C4—H4	0.9500	C27—H27B	0.9900
C5—C6	1.399 (6)	C28—C29	1.453 (7)
C5—H5	0.9500	C28—H28A	0.9900
C7—C8	1.519 (6)	C28—H28B	0.9900
C8—C9	1.519 (6)	C29—C30	1.569 (7)
C8—C24	1.535 (6)	C29—H29A	0.9900
C8—H8	1.0000	C29—H29B	0.9900
C10—C11	1.520 (6)	C30—C31	1.508 (7)
C10—H10A	0.9900	C30—H30A	0.9900
C10—H10B	0.9900	C30—H30B	0.9900
C11—C16	1.382 (6)	C31—C32	1.520 (7)
C11—C12	1.395 (6)	C31—H31A	0.9900
C12—C13	1.389 (6)	C31—H31B	0.9900
C12—H12	0.9500	C32—C33	1.541 (7)
C13—C14	1.382 (8)	C32—H32A	0.9900
C13—H13	0.9500	C32—H32B	0.9900
C14—C15	1.376 (8)	C33—C34	1.462 (9)
C14—H14	0.9500	C33—H33A	0.9900
C15—C16	1.382 (7)	C33—H33B	0.9900
C15—H15	0.9500	C34—H34A	0.9800
C16—H16	0.9500	C34—H34B	0.9800
C17—C18	1.512 (6)	C34—H34C	0.9800
			
C9—N1—C1	124.2 (3)	C20—C19—H19	119.4
C9—N1—C10	117.5 (4)	C18—C19—H19	119.4
C1—N1—C10	118.3 (4)	C19—C20—C21	119.2 (6)
C7—N2—C6	122.8 (3)	C19—C20—H20	120.4
C7—N2—C17	118.4 (3)	C21—C20—H20	120.4
C6—N2—C17	118.1 (3)	C22—C21—C20	119.8 (6)
N4—N3—C25	109.0 (4)	C22—C21—H21	120.1
N3—N4—N5	107.1 (4)	C20—C21—H21	120.1
N4—N5—C26	110.8 (4)	C21—C22—C23	120.8 (6)
N4—N5—C27	119.8 (5)	C21—C22—H22	119.6
C26—N5—C27	129.4 (5)	C23—C22—H22	119.6
C2—C1—C6	119.0 (4)	C22—C23—C18	121.3 (6)
C2—C1—N1	119.2 (4)	C22—C23—H23	119.4
C6—C1—N1	121.7 (4)	C18—C23—H23	119.4
C3—C2—C1	120.8 (4)	C25—C24—C8	113.0 (4)
C3—C2—H2	119.6	C25—C24—H24A	109.0
C1—C2—H2	119.6	C8—C24—H24A	109.0
C4—C3—C2	120.2 (5)	C25—C24—H24B	109.0
C4—C3—H3	119.9	C8—C24—H24B	109.0
C2—C3—H3	119.9	H24A—C24—H24B	107.8
C3—C4—C5	120.1 (4)	N3—C25—C26	107.5 (4)
C3—C4—H4	119.9	N3—C25—C24	122.2 (4)
C5—C4—H4	119.9	C26—C25—C24	130.2 (4)
C4—C5—C6	120.8 (4)	N5—C26—C25	105.5 (4)
C4—C5—H5	119.6	N5—C26—H26	127.2
C6—C5—H5	119.6	C25—C26—H26	127.2
C5—C6—C1	119.0 (4)	N5—C27—C28	114.8 (5)
C5—C6—N2	119.0 (4)	N5—C27—H27A	108.6
C1—C6—N2	121.9 (4)	C28—C27—H27A	108.6
O2—C7—N2	122.2 (4)	N5—C27—H27B	108.6
O2—C7—C8	121.7 (4)	C28—C27—H27B	108.6
N2—C7—C8	116.1 (4)	H27A—C27—H27B	107.5
C7—C8—C9	105.1 (4)	C29—C28—C27	115.7 (6)
C7—C8—C24	110.8 (4)	C29—C28—H28A	108.4
C9—C8—C24	112.3 (4)	C27—C28—H28A	108.4
C7—C8—H8	109.5	C29—C28—H28B	108.4
C9—C8—H8	109.5	C27—C28—H28B	108.4
C24—C8—H8	109.5	H28A—C28—H28B	107.4
O1—C9—N1	122.4 (4)	C28—C29—C30	113.3 (6)
O1—C9—C8	122.6 (4)	C28—C29—H29A	108.9
N1—C9—C8	115.0 (4)	C30—C29—H29A	108.9
N1—C10—C11	116.0 (4)	C28—C29—H29B	108.9
N1—C10—H10A	108.3	C30—C29—H29B	108.9
C11—C10—H10A	108.3	H29A—C29—H29B	107.7
N1—C10—H10B	108.3	C31—C30—C29	115.4 (5)
C11—C10—H10B	108.3	C31—C30—H30A	108.4
H10A—C10—H10B	107.4	C29—C30—H30A	108.4
C16—C11—C12	118.9 (4)	C31—C30—H30B	108.4
C16—C11—C10	123.5 (4)	C29—C30—H30B	108.4
C12—C11—C10	117.6 (4)	H30A—C30—H30B	107.5
C13—C12—C11	120.1 (5)	C30—C31—C32	113.0 (5)
C13—C12—H12	119.9	C30—C31—H31A	109.0
C11—C12—H12	119.9	C32—C31—H31A	109.0
C14—C13—C12	120.4 (5)	C30—C31—H31B	109.0
C14—C13—H13	119.8	C32—C31—H31B	109.0
C12—C13—H13	119.8	H31A—C31—H31B	107.8
C15—C14—C13	119.2 (5)	C31—C32—C33	112.1 (5)
C15—C14—H14	120.4	C31—C32—H32A	109.2
C13—C14—H14	120.4	C33—C32—H32A	109.2
C14—C15—C16	120.9 (5)	C31—C32—H32B	109.2
C14—C15—H15	119.5	C33—C32—H32B	109.2
C16—C15—H15	119.5	H32A—C32—H32B	107.9
C15—C16—C11	120.4 (5)	C34—C33—C32	112.2 (6)
C15—C16—H16	119.8	C34—C33—H33A	109.2
C11—C16—H16	119.8	C32—C33—H33A	109.2
N2—C17—C18	113.5 (3)	C34—C33—H33B	109.2
N2—C17—H17A	108.9	C32—C33—H33B	109.2
C18—C17—H17A	108.9	H33A—C33—H33B	107.9
N2—C17—H17B	108.9	C33—C34—H34A	109.5
C18—C17—H17B	108.9	C33—C34—H34B	109.5
H17A—C17—H17B	107.7	H34A—C34—H34B	109.5
C23—C18—C19	117.7 (5)	C33—C34—H34C	109.5
C23—C18—C17	121.1 (4)	H34A—C34—H34C	109.5
C19—C18—C17	121.1 (4)	H34B—C34—H34C	109.5
C20—C19—C18	121.2 (6)		
			
C25—N3—N4—N5	0.3 (7)	N1—C10—C11—C16	−5.8 (7)
N3—N4—N5—C26	0.7 (6)	N1—C10—C11—C12	175.1 (4)
N3—N4—N5—C27	−177.2 (5)	C16—C11—C12—C13	0.4 (7)
C9—N1—C1—C2	135.7 (5)	C10—C11—C12—C13	179.5 (4)
C10—N1—C1—C2	−44.3 (6)	C11—C12—C13—C14	−1.2 (8)
C9—N1—C1—C6	−45.7 (6)	C12—C13—C14—C15	0.6 (8)
C10—N1—C1—C6	134.4 (4)	C13—C14—C15—C16	0.9 (9)
C6—C1—C2—C3	−1.3 (7)	C14—C15—C16—C11	−1.6 (8)
N1—C1—C2—C3	177.3 (5)	C12—C11—C16—C15	1.0 (7)
C1—C2—C3—C4	0.5 (8)	C10—C11—C16—C15	−178.1 (5)
C2—C3—C4—C5	0.8 (8)	C7—N2—C17—C18	−127.1 (4)
C3—C4—C5—C6	−1.2 (7)	C6—N2—C17—C18	62.2 (5)
C4—C5—C6—C1	0.3 (7)	N2—C17—C18—C23	−121.0 (4)
C4—C5—C6—N2	−177.2 (5)	N2—C17—C18—C19	62.2 (6)
C2—C1—C6—C5	1.0 (7)	C23—C18—C19—C20	−1.9 (8)
N1—C1—C6—C5	−177.7 (4)	C17—C18—C19—C20	175.0 (5)
C2—C1—C6—N2	178.4 (4)	C18—C19—C20—C21	0.5 (10)
N1—C1—C6—N2	−0.3 (7)	C19—C20—C21—C22	1.3 (10)
C7—N2—C6—C5	−132.9 (5)	C20—C21—C22—C23	−1.7 (10)
C17—N2—C6—C5	37.4 (6)	C21—C22—C23—C18	0.2 (9)
C7—N2—C6—C1	49.7 (6)	C19—C18—C23—C22	1.6 (8)
C17—N2—C6—C1	−140.0 (4)	C17—C18—C23—C22	−175.3 (5)
C6—N2—C7—O2	175.4 (4)	C7—C8—C24—C25	−171.2 (4)
C17—N2—C7—O2	5.1 (7)	C9—C8—C24—C25	71.6 (5)
C6—N2—C7—C8	−7.1 (6)	N4—N3—C25—C26	−1.0 (7)
C17—N2—C7—C8	−177.3 (4)	N4—N3—C25—C24	175.9 (5)
O2—C7—C8—C9	106.5 (5)	C8—C24—C25—N3	66.7 (6)
N2—C7—C8—C9	−71.1 (5)	C8—C24—C25—C26	−117.1 (6)
O2—C7—C8—C24	−15.0 (6)	N4—N5—C26—C25	−1.3 (6)
N2—C7—C8—C24	167.4 (4)	C27—N5—C26—C25	176.3 (5)
C1—N1—C9—O1	177.8 (4)	N3—C25—C26—N5	1.4 (6)
C10—N1—C9—O1	−2.3 (6)	C24—C25—C26—N5	−175.2 (5)
C1—N1—C9—C8	−0.3 (6)	N4—N5—C27—C28	−73.6 (7)
C10—N1—C9—C8	179.7 (3)	C26—N5—C27—C28	109.0 (7)
C7—C8—C9—O1	−102.6 (5)	N5—C27—C28—C29	−56.3 (10)
C24—C8—C9—O1	17.9 (6)	C27—C28—C29—C30	−166.2 (6)
C7—C8—C9—N1	75.4 (5)	C28—C29—C30—C31	−58.8 (10)
C24—C8—C9—N1	−164.0 (4)	C29—C30—C31—C32	−166.2 (6)
C9—N1—C10—C11	−80.1 (5)	C30—C31—C32—C33	−172.4 (6)
C1—N1—C10—C11	99.9 (5)	C31—C32—C33—C34	170.1 (6)
